# Respiratory support strategies in the prevention of bronchopulmonary dysplasia: A single center quality improvement initiative

**DOI:** 10.3389/fped.2022.1012655

**Published:** 2022-12-12

**Authors:** Heather White, Kamaris Merritt, Kirsti Martin, Emily Lauer, Lawrence Rhein

**Affiliations:** ^1^ Division of Neonatology, Department of Pediatrics, University of Massachusetts Memorial Medical Center, Worcester, MA, United States; ^2^ Department of Family Medicine and Community Health, University of Massachusetts Chan Medical School, Worcester, MA, United States; ^3^ Eunice Kennedy Shriver Center, University of Massachusetts Chan Medical School, Worcester, MA, United States

**Keywords:** bronchopulmonar dysplasia, surfactant, caffeine, non-invasive ventilation (NIV), quality improvement - outcomes

## Abstract

**Background and objectives:**

Bronchopulmonary dysplasia (BPD) continues to be a significant morbidity affecting very preterm infants, despite multiple advancements in therapies to treat respiratory distress syndrome and prevent BPD. Local quality improvement (QI) efforts have shown promise in reducing unit or system-wide rates of BPD. In preterm infants born between 23- and 32-weeks' gestation, our aim was to decrease the rate of BPD at 36 weeks corrected gestational age from 43% to 28% by January 2019.

**Methods:**

Directed by a multidisciplinary respiratory QI team, we gradually implemented the following interventions to reach our aim: (1) early initiation of non-invasive ventilation in the delivery room, (2) initiation of caffeine prior to 24 h of life, (3) administration of early selective surfactant per a well-defined guideline, (4) continuation of non-invasive ventilation until 32 and 0/7 weeks corrected gestational age (CGA), and (5) a revision of the early selective surfactant guideline. Outcome measures included rates of BPD, and process measures included compliance with the above interventions.

**Results:**

A total of 509 infants with an average gestational age of 29 1/7 weeks and birth weight of 1,254 (SD±401) grams were included. The rate of BPD in our unit decreased from a baseline of 43% to 19% from the start of the project in October 2016 until the first quarter of 2022 (*p* < 0.00001). The greatest reductions in BPD rates were seen after the initiation of the guideline to extend non-invasive ventilation until 32 0/7 weeks CGA. The rate of severe BPD decreased from 22% to 9%.

**Conclusions:**

In preterm infants born between 23- and 32-weeks' gestation, our local QI interventions to reduce rates of BPD were associated with a reduction in rates by 56%. Increased use of antenatal steroids and higher birth weights post- vs. pre-intervention may have contributed to this successes.

## Introduction

Over the last two decades, survival without significant morbidity among very low birthweight (VLBW) infants has improved (1, 2). Steady decreases in the rate of morbidities such as severe intraventricular hemorrhage, retinopathy of prematurity, late onset sepsis, and necrotizing enterocolitis have been observed, yet rates of bronchopulmonary dysplasia (BPD) have remained consistent (1–3). Despite critical advances in treating respiratory distress syndrome (RDS) and BPD, almost half of surviving extremely preterm infants will develop BPD. The most widely accepted definition for BPD is an oxygen requirement at 36 weeks corrected gestational age (CGA) (4, 5).

While there has been extensive research regarding individual treatment strategies for preventing or reducing BPD, including use of caffeine (6, 7), early non-invasive respiratory support (8), and early selective surfactant administration (9), information regarding how individual neonatal intensive care units (NICUs) can implement these therapies to optimize local outcomes has only recently begun to emerge, in the form of published Quality Improvement (QI) efforts (10). Continued description of QI efforts may provide other NICUs with new ideas and strategies they can use in the quest to prevent and reduce BPD in their own unit.

In this article, we will describe the gradual implementation of the components of our unit's BPD Prevention Quality Improvement Bundle, focusing on optimizing our delivery room (DR) respiratory support strategies and early NICU course management. The initial intervention was a “DR Bundle” consisting of early initiation of non-invasive ventilation in the DR, initiation of caffeine prior to 24 h of life, and administration of early selective surfactant per a well-defined guideline. Subsequent interventions included continuation of non-invasive ventilation until 32 and 0/7 weeks corrected gestational age (CGA) and a revision of the early selective surfactant guideline. This initiative was conducted at The University of Massachusetts Chan Medical School and UMass Memorial Medical Center (UMMMC) NICU.

### Aims

**The** goal of our initiative was to optimize our DR resuscitation guidelines and early neonatal course management for infants born at 32 0/7 weeks gestation or less, in order to decrease the incidence of BPD in our unit. During the UMMMC NICU's annual review of respiratory outcomes in late 2016, our BPD committee and QI team evaluated the respiratory management and BPD incidence rates of infants born ≤32 0/7 weeks previously admitted to our unit. At 36 0/7 weeks corrected gestational age (CGA), the BPD rate for infants born ≤32 0/7 was 43%, with rates in some quarters (3-month intervals) over 50%. Our team defined BPD as an oxygen requirement at 36 0/7 weeks CGA using the National Institute of Child Health and Human Development/National Heart, Lung and Blood Institute (NICHD/NHLBI) 2001 definition (4). The global aim of our ongoing respiratory QI initiative is to improve respiratory outcomes for preterm infants by decreasing our unit's BPD rate by 35%. Key drivers for this global aim are illustrated in the key driver diagram shown in Supplementary Figure S1.

As the first step in attempting to reach our global aim, our QI team targeted a SMART Aim to increase our compliance with our BPD Bundle guideline elements at birth to minimize lung injury from 43% to 90% by January 1, 2018. We set outcome measure goals for each of our interventions, keeping track of compliance with each intervention. Balancing measures of pneumothoraxes and ventilator utilization were also monitored during the project.

## Methods

UMMMC is a 49-bed Level III NICU in Central Massachusetts with approximately 4500 deliveries and 650 admissions annually. The NICU serves as the only tertiary care center in Central Massachusetts and runs an active neonatal transport program serving this region. The NICU admits approximately 100 infants born under 32 0/7 weeks and 120 very low birth weight (VLBW) infants annually. The UMMMC NICU is in an academic center with a multidisciplinary team consisting of 7 neonatologists, 12 neonatal nurse practitioners, 1 physician assistant, 3 neonatal fellows, 2–4 rotating resident physicians per month, 7 respiratory therapists, 1 neonatal pharmacist, 1 full-time neonatal dietician, and 150 staff nurses that participate in carrying out the respiratory management of infants in the NICU. To change practice and sustain change over time, we therefore estimate that the number needed to influence is approximately 200 staff members. This work was reviewed by the Institutional Review Board of the UMass Chan Medical School and was determined to be exempt from full IRB review.

In October 2016, a multidisciplinary respiratory QI team was formed to assess the state of respiratory practice in the UMMMC NICU. Access to specific respiratory guidelines for infants born ≤32 0/7 weeks gestation prior to this time were not available. The team specifically tailored data collection tools to determine our unit's baseline BPD rate and to identify areas of potential focus.

In January 2017, a “Minimizing Lung Injury” delivery room bundle was introduced, which included (a) guidelines for early initiation of non-invasive ventilation in the DR, in order to avoid intubation for mechanical ventilation, (b) early surfactant administration for qualifying infants, and (c) early administration of caffeine to prevent apnea of prematurity (Supplementary Figure S2).

Per the DR Bundle flow diagram, NICU providers were instructed to initially place infants born between 23 0/7 and 26 6/7 weeks on noninvasive positive pressure ventilation (NIPPV) *via* NeoTech RAM cannula (NeoTech Products, Valencia, CA, United States) at a positive inspiratory pressure (PIP) of 25 centimeters of water (cm H_2_0), positive end expiratory pressure (PEEP) of 7 cm H_2_0, and rate of 30 breaths per minute within 2 min of life. Infants born between 27 0/7 and 32 0/7 weeks were to be placed on continuous positive airway pressure (CPAP) *via* RAM cannula at PEEP of 6 to 8 cm H_2_0 within 2 min of life. If infants showed signs of increased work of breathing, apnea, or bradycardia, a trial of NIPPV was attempted, to avoid intubation for mechanical ventilation. Non-invasive ventilation was delivered *via* a NeoPuff T-piece resuscitator (Fisher & Paykel Healthcare, Auckland, New Zealand). Infants who required intubation in the DR due to lack of respiratory effect per the Neonatal Resuscitation Protocol were to be given surfactant within two hours of life. Infants who were maintained on non-invasive ventilation who had an FiO_2_ requirement of greater than 40%, significant increased work of breathing with moderate to severe retractions and/or respiratory rate >80 times per minute were to receive surfactant *via* the INSURE method by 90 min of life. All infants ≤32 0/7 weeks were ordered to start caffeine at a dose of 20 mg/kg and were then maintained on daily caffeine doses of 10 mg/kg. Doses were given intravenously until infants were advanced to full enteral feeds and no longer had IV access.

We initiated these bundle guidelines *via* several plan-do-study-act (PDSA) cycles, including placing a large poster of the DR Bundle in the resuscitation room outside of the delivery/operating room, as well as provider and respiratory therapist training on T-piece resuscitator use. In conjunction with the roll out of a new electronic medical record system in October 2017, order sets for caffeine were implemented to ensure compliance with the early caffeine guideline. Since the interventions were focused on developing protocols, all PDSAs involved our multidisciplinary BPD committee members and were tested by all key disciplines for feedback prior to initiation and during implementation. We set outcome measure goals to have 90% compliance with each element of the DR bundle by January 1, 2018.

After further data collection and QI work, our BPD committee reconvened in December 2017 to review our respiratory outcomes. Although we noted improvements in the BPD rate, our NICU still had not reached our SMART aim of reducing BPD rates by 35%. In January 2018, we implemented a new guideline advising that all infants born <32 0/7 weeks gestation remain on non-invasive ventilation until at least 32 0/7 weeks CGA. This guideline was based on animal models showing that CPAP and resultant functional residual capacity (FRC) maintained by mechanical lung distention promoted lung development by providing stretch to the pulmonary tissue, and important stimulus for lung growth (11). We hoped that this strategy would not only promote further lung growth in our population, but also maintain FRC to prevent alveolar atelectasis that otherwise may have necessitated an escalation of respiratory support once CPAP was removed. This guideline was subsequently supported by the results of a study by Lam et al. which showed a greater increase in FRC after two weeks of continued CPAP and at hospital discharge for infants who were randomized to extended CPAP when compared to those randomized to usual CPAP discontinuation (12). We set outcome measure goals to have 90% compliance with continued non-invasive ventilation by January 1, 2019.

In January 2020, an internal audit on our unit's surfactant utilization and timing of administration identified that improvements could be made in identifying infants who would likely benefit from early selective surfactant and administering surfactant prior to two hours of life. At this time, we took on another PDSA cycle, developing a guideline aimed at early identification of these infants and the timely administration of surfactant. Criteria for early identification and administration of surfactant included assessment of each infant's gestational age, respiratory support in the DR and at 90 min of age (including modality and pressure used), FiO2 requirement, and physical exam/lab findings concerning for significant respiratory distress (Supplementary Figure S3). Infants who required intubation in the DR for resuscitation were to be given surfactant while they were intubated unless the need for intubation was deemed to be due to respiratory depression attributable to other factors such as maternal magnesium infusion. Surfactant was to be delivered *via* the INSURE method for infants who remained on non-invasive ventilation but met criteria for early selective surfactant administration (13). This guideline was implemented starting in August 2020. We set outcome measure goals to have 90% compliance with our new early selective surfactant guideline by August 1, 2021.

### Measures

Clinical data was abstracted from the infants' electronic medical records, including respiratory support required in the delivery room, timing of first caffeine administration, timing of surfactant administration, days of invasive mechanical ventilation, last day of non-invasive ventilation, post-natal steroid administration, BPD status, presence of pneumothorax, length of stay, corrected gestational age at discharge, and discharge home on oxygen therapy. Demographic data was also collected, including gender, birth weight, gestational age, race/ethnicity, maternal age, and antenatal steroid status. Data for outborn infants was collected but excluded for purposes of this analysis due to inability of these infants to consistently receive our DR Bundle at delivery. Infants who did not survive to 36 weeks CGA were also excluded from the analysis. Data was collected monthly for each infant and analyzed by quarter (3-month interval).

The primary outcome measure was the percentage of inborn infants born ≤32 0/7 who developed moderate or severe BPD per the NICHD/NHLBI 2001 consensus definition (infants requiring oxygen or positive pressure at 36 weeks CGA) (4, 5). Infants with mild BPD were not included as having BPD in this measure, as these infants did not require supplemental oxygen at 36 weeks CGA. A process control chart was utilized to track changes in the BPD rate in relation to the interventions throughout the project period.

Process measures included compliance with DR bundle interventions (measured separately as receipt of early non-invasive respiratory support in the delivery room, administration of caffeine prior to 24 h of life, and administration of early selective surfactant at less than two hours of life), compliance with continued CPAP until 32 0/7 weeks PMA, and compliance with the August 2020 revised early selective surfactant guideline. For the purposes of this analysis, infants who continued CPAP until 31 6/7 weeks CGA were considered compliant as infants often were removed from CPAP during evening rounds, within 4 h of turning 32 0/7 CGA. Overall compliance with the BPD Prevention QI bundle was also measured; compliance with protocol definitions changed with each additional guideline and PDSA cycle. Compliance rates for each guideline were analyzed using *P* charts for data involving classification of compliant vs. non-compliant. The balancing measure of days of invasive mechanical ventilation by patient were analyzed using a C chart. The balancing measure of pneumothorax rate was not placed into a *P* chart due to its low occurrence. Charts were prepared using Macros (KnowWare International, Inc, Denver, CO, United States) add-on in Microsoft Excel (Microsoft Corporation, Redmond, WA, United States) to display and analyze data over time. The center line (CL) was defined as the running average for 8-consecutive points on the same side of the CL, or successive increasing or decreasing points. The upper control limit (UCL) and lower control limit (LCL) define two of three points beyond 2 standard deviations from the mean on the same side of the CL (14).

Data was analyzed in four columns representing the four epochs before and after implementation of QI measures. Differences between groups on key demographic factors of mothers and infants were assessed with Friedman ANOVAs as shown in Table 1. Any demographics showing significant differences (*p* < 0.05) between groups were then analyzed with either Chi-squared or Fisher's exact tests for categorical variables, or post-hoc Mann Whitney *U* tests for continuous variables. Similarly, the association between groups and outcomes of BPD, death, death or BPD, invasive mechanical ventilation, postnatal steroid use and pneumothorax were first analyzed with Friedman ANOVAs as shown in Table 2. Specific comparisons between each set of groups were then assessed with the same tests as the demographic variables. In addition, logistic regressions were used selectively to look at the relationships between key findings based on the outcome of the group comparisons to test whether the group association with BPD as an outcome changed when controlling for particular factors as described in results. Bivariate and multivariable statistics were calculated with SAS 9.4 (Cary, NC, United States).

**Table 1:** Cohort demographics Pre- and post-quality improvement initiative implementation.

	Pre-Delivery Room Bundle	Post-Delivery Room Bundle and Other QI Interventions			
**Baseline** (Q1 2016-Q4 2016) *N* = 86	**DR Bundle Implementation** (Q1 2017-Q4 2017) *N* = 109	**CPAP Until 32 0/7 CGA** (Q1 2018- Q2 2020) *N* = 249	**Surfactant Guideline Revision** (Q3 2020-Q1 2021) *N* = 167		
*N* (%)				*p*-Value
**Maternal Characteristics**
Chorioamnionitis		3 (4)	2 (2)	8 (3)	10 (6)	0.1239
Hypertension		40 (47)	41 (38)	70 (28)	56 (34)	*0.0497
Diabetes Mellitus		10 (12)	9 (8)	34 (14)	28 (17)	0.0664
Delivery Mode						
Vaginal Delivery		32 (37)	37 (34)	83 (33)	54 (32)	0.4895
C-section		54 (63)	72 (66)	166 (67)	113 (68)	
Antenatal Steroids						
Full course		49 (57)	59 (54)	152 (61)	110 (66)	*0.0012
Partial course		16 (19)	26 (24)	77 (31)	39 (23)	
No steroids		21 (24)	24 (22)	20 (8)	18 (11)	
**Infant Characteristics**						
Gender, Male		50 (58)	57 (52)	129 (52)	91 (55)	0.8008
Ethnicity						
Hispanic Latino		19 (22)	22 (20)	53 (21)	46 (28)	0.0074
Not specified		2 (2)	4 (4)	0 (0)	0 (0)	
Race						
Asian		1 (1)	4 (4)	14 (6)	9 (6)	0.1426
Black or African American		12 (14)	14 (13)	34 (14)	24 (14)	0.8097
White		62 (72)	72 (66)	158 (63)	107 (64)	0.2675
Inborn		76 (88)	100 (92)	228 (92)	159 (95)	0.0675
Outborn		10 (12)	9 (8)	21 (8)	8 (5)	
Death		8 (9)	10 (9)	25 (10)	18 (11)	0.6317
Maternal Age (yrs)	Median	30	30	31	31	
	Mean	29.8	30.6	30.3	30.9	
	Std. Dev	5.5	6.1	5.5	6.1	
Gestational Age (wks)	Median	29.3	29.3	29.7	29.4	
	Mean	28.5	28.9	28.9	29.0	
	Std. Dev	2.7	2.3	2.6	2.5	
Infant Birthweight (g)	Median	1120	1225	1263.5	1340	*0.0055
	Mean	1168	1221.8	1257.7	1320.1	
** **	Std. Dev	442.2	357.3	401.9	401.2	

* *p*-value of ≤0.05 was considered significantly significant.

No significant differences between groups in Mann–Whitney *U* test (two-sided *p*-value).

Chi squared test used for Ethnicity, hypertension and antenatal steroids given variable structure.

**Table 2:** Respiratory outcomes Pre- and post-quality improvement initiative implementation.

		Pre-Delivery Room Bundle	Post-Delivery Room Bundle and Other QI Interventions			
		**Baseline** (Q1 2016-Q4 2016) *N* = 86	**DR Bundle Implementation** (Q1 2017- Q4 2017) *N* = 109	**CPAP Until 32 0/7 CGA** (Q1 2018- Q2 2020) *N* = 249	**Surfactant Guideline Revision** (Q3 2020-Q1 2021) *N* = 167	
		*N (%)*				*p*-Value
Moderate to Severe BPD		33 (38)	29 (27)	58 (23)	25 (15)	<0.0001*
Death		8 (9)	10 (9)	25 (10)	18 (11)	0.6317
Composite Moderate to Severe BPD or Death		41 (48)	39 (36)	83 (33)	43 (26)	0.0009*
Required Invasive Mechanical Ventilation		45 (52)	26 (24)	43 (17)	45 (27)	0.0025*
Postnatal Steroids		16 (19)	21 (19)	36 (14)	17 (10)	0.0212*
Pneumothorax		1 (1)	7 (6)	10 (4)	5 (3)	0.8320
Corrected Gestational Age Positive Pressure Removed	Median	32	32	32	32.1	
	Mean	32.4	32.5	32.8	32.9	
	Std. Dev	2.1	1.5	1.8	1.8	

* *p*-value of ≤0.05 was considered significantly significant.

BPD Status was defined using the NICHD criteria for moderate and severe BPD.

## Results

A total of 611 infants were born between 23 0/7 and 32 0/7 weeks gestational age (GA) and admitted to the UMMMC NICU during the baseline and PDSA cycle periods. Cohort demographics are presented in Table 1. Significant differences included lower instances of maternal hypertension and higher utilization of full course antenatal steroids in later cohorts compared to baseline. Despite significance in group differences in an overall ANOVA test, Chi-squared tests for specific group differences did not indicate any significant differences in the number of Hispanic infants between specific groups. There was only a significant difference in median birth weights between the second intervention group (CPAP Until 32 CGA) and baseline (*p* = 0.0055). All other group comparisons on birthweights did not show differences in Mann Whitney *U* tests.

**Figure 1:**
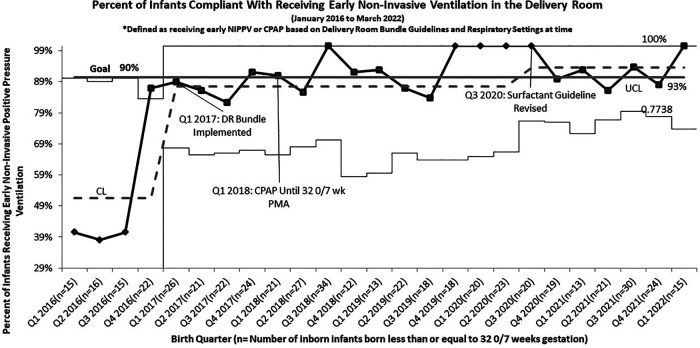
*P* chart of compliance with delivery room bundle for infants born less than or equal to 32 0/7 weeks gestation who received early continuous Non-invasive positive pressure by guidelines.

**Figure 2:**
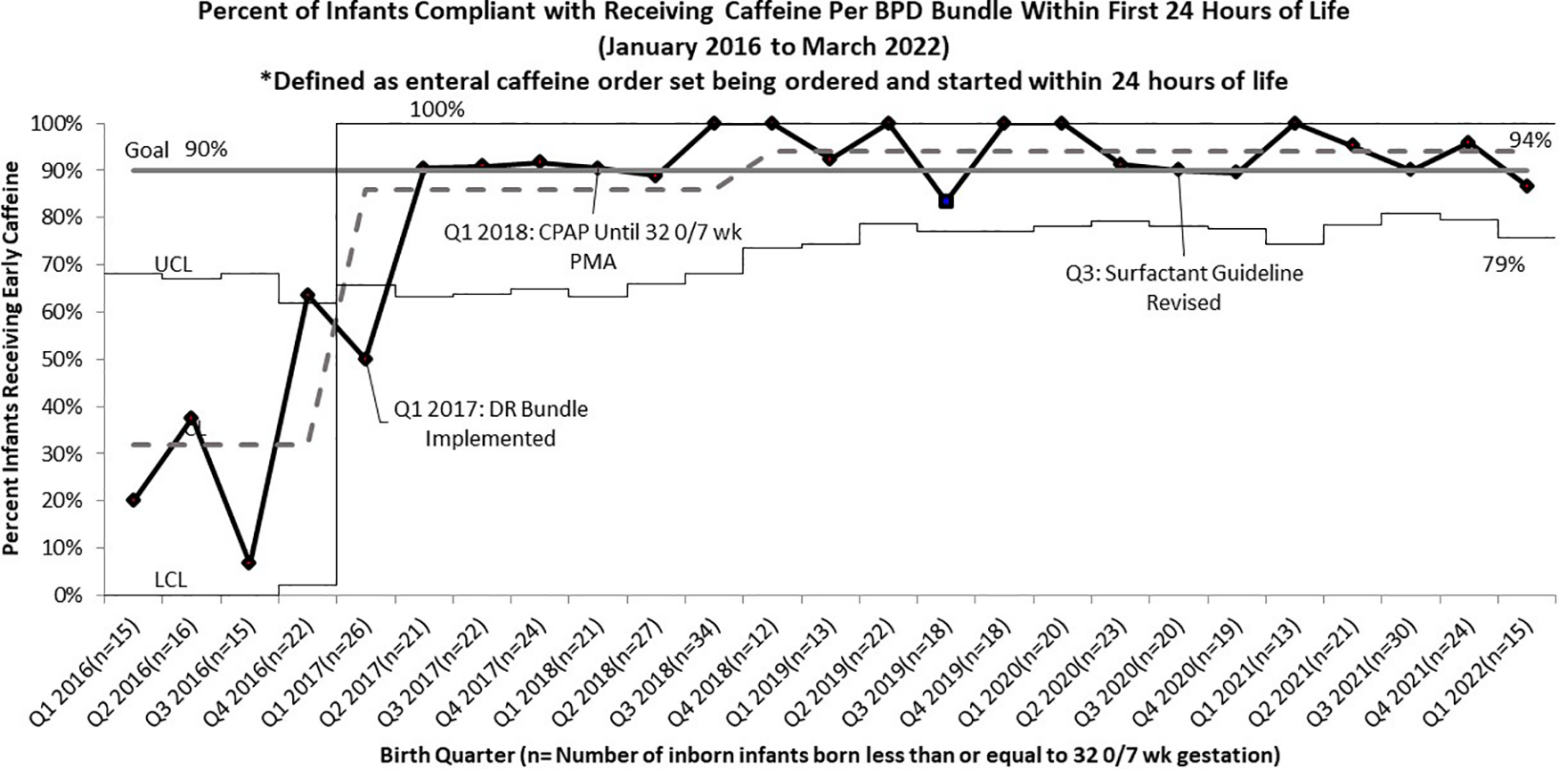
*P* chart of compliance with BPD bundle guidelines for infants born less than or equal to 32 0/7 Weeks Gestation to Receive Early Caffeine Within 24 h of life.

Following the roll out of the DR Bundle in January 2017, the rate of infants receiving early non-invasive ventilation in the delivery room quickly increased from a baseline of 51% to 87% (Figure 1). Further increases in compliance with this guideline were seen after four straight quarters of 100% compliance from Q4 2019 to Q3 2020, allowing the percent compliance to increase to 93%, past our goal of 90%. Compliance with initiation of caffeine within 24 h of life as part of this DR bundle saw similar improvements, increasing from a pre-intervention baseline of 32% to 86%. Compliance with this guideline increased past the goal of 90% to 94% in the quarters following the initiation of continued non-invasive ventilation until 32 0/7 weeks PMA (Figure 2).

**Figure 3:**
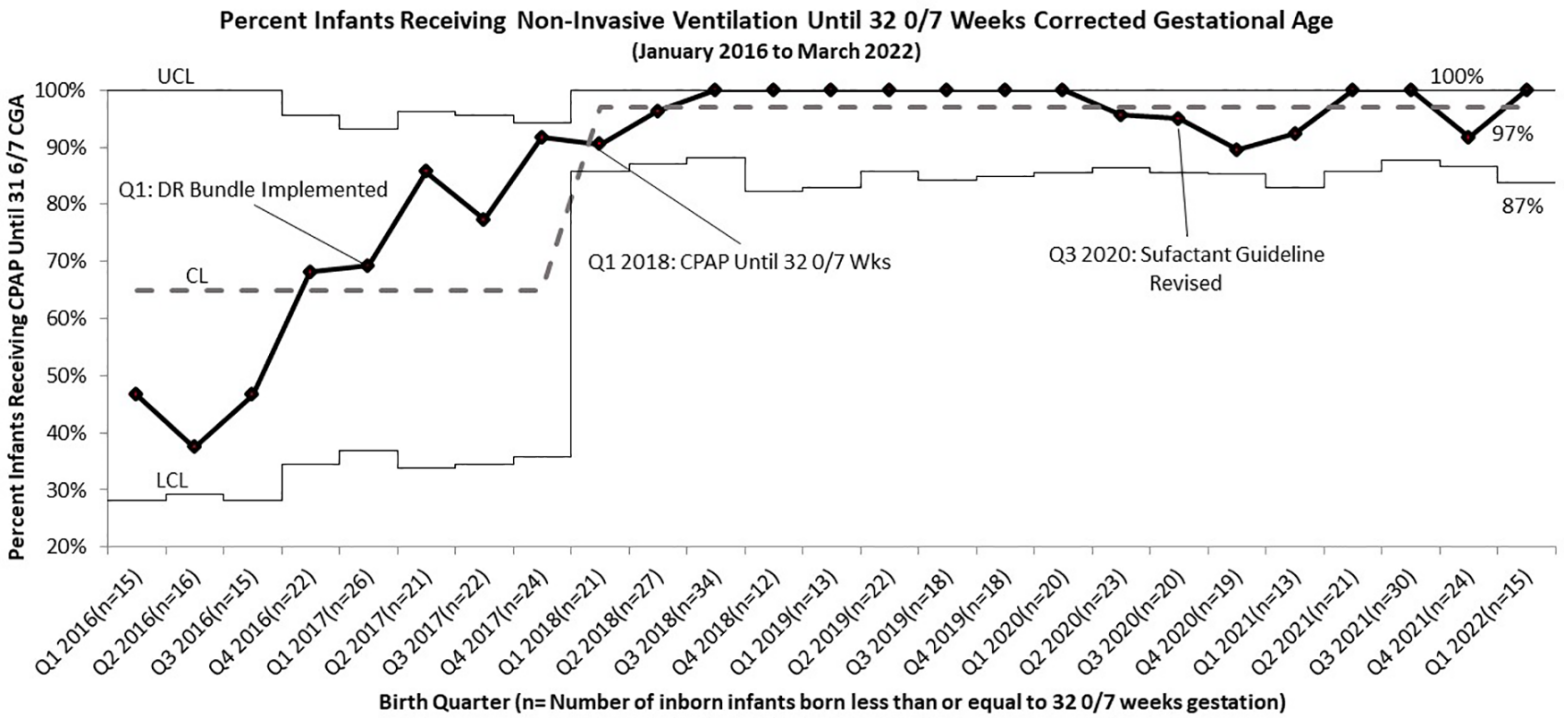
*p* Chart of Compliance with BPD Bundle Guidelines for Infants Born Less than or Equal to 32 0/7 Weeks Gestation to Receive Continuous Positive Airway Pressure Until 32 0/7 Weeks Corrected Gestational Age (Including those that were removed the evening of 31 6/7 Weeks).

Following PDSA cycle evaluations for BPD rates after our initial DR bundle intervention, we implemented the continued non-invasive ventilation until 32 0/7 weeks CGA guideline. Compliance rates for this guideline increased from 55% to 83% after implementation. After adjusting for infants who were taken off of non-invasive ventilation on night rounds at 31 6/7 weeks, just hours from turning 32 0/7, compliance increased to 97%, surpassing the goal of 90% (Figure 3).

Compliance with the guideline for early selective surfactant that was rolled out as part of the DR bundle in January 2017 was tracked as receipt of surfactant prior to two hours of life; just 25% of infants were receiving surfactant at less than 2 h of life from Q1 2016 to Q2 2020. The new early selective surfactant guideline was created and implemented in Q3 2020. A baseline rate of compliance with this new guideline was collected retrospectively from Q4 of 2019 to Q2 of 2020 and was determined to be 36%. Implementation of this new guideline resulted in an increase in compliance with the guideline beyond 85%. It also resulted in an increase in administration of surfactant less than two hours to 51% of infants who received surfactant since August 2020 (38 of 74 infants; 137 total infants born during this time), compared to 36% of infants receiving surfactant in 2019.

**Figure 4:**
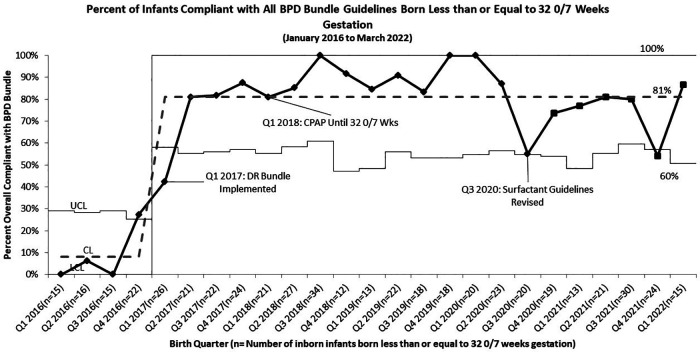
*p* chart of compliance with all BPD bundle guidelines for infants born less than or equal to 32 0/7 weeks gestation.

**Figure 5:**
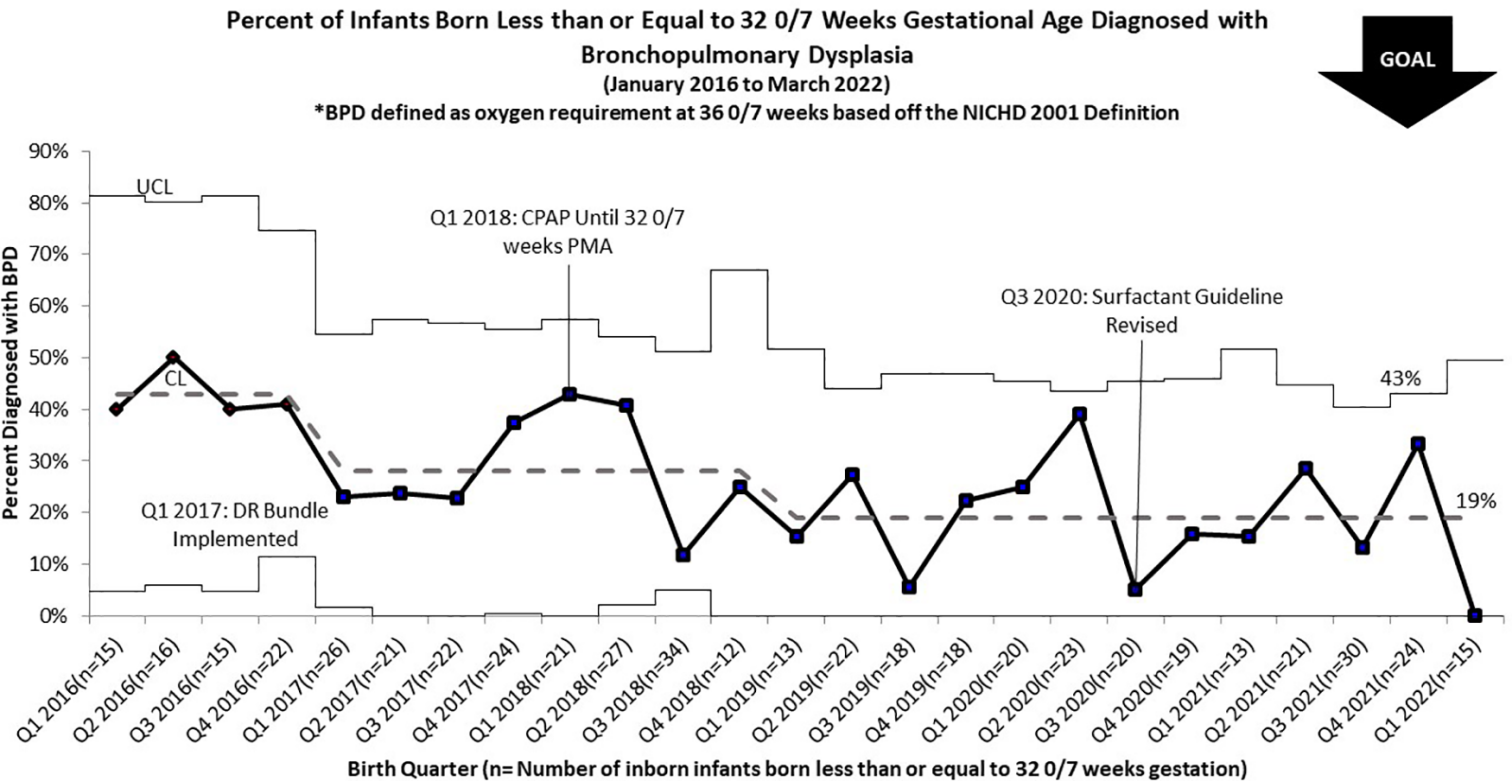
*p* chart for percent of infants born less than or equal to 32 0/7 weeks gestation diagnosed with BPD by NICHD criteria.

**Figure 6:**
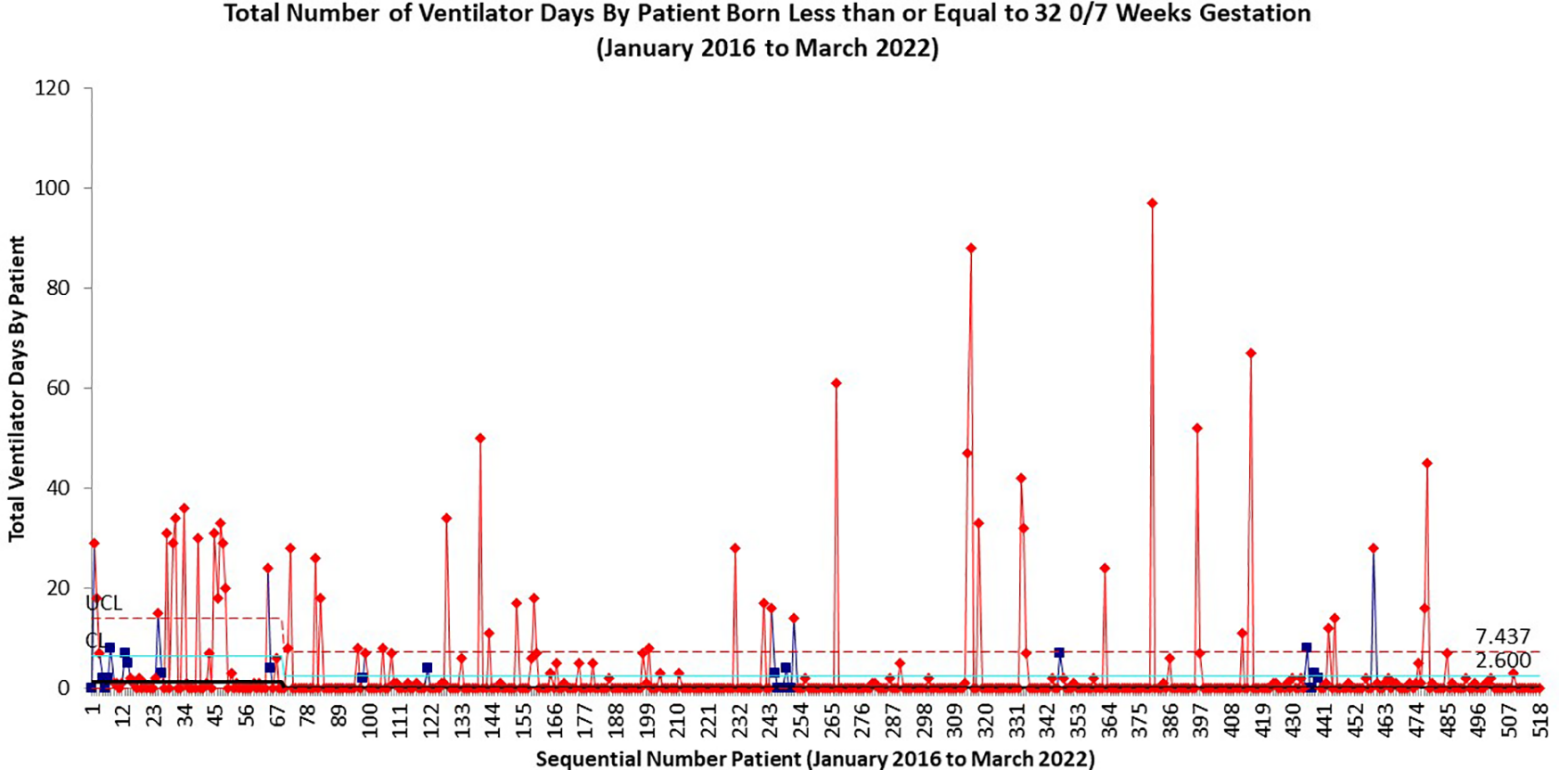
Ventilator days for infants born less than or equal to 32 0/7 weeks gestation.

Overall compliance with all aspects of our sequentially integrated guidelines improved from 9% at baseline to 81% at closeout (Figure 4). The pre-intervention cohort rate of BPD was 43%, which was utilized as the baseline unit rate of BPD. After implementation of the DR Bundle in January 2017 and continued non-invasive ventilation until 32 0/7 weeks CGA, unit-wide BPD rates decreased to a midpoint of 28%. Combined with these interventions, implementation of the revised early selective surfactant guideline in Q3 of 2020 saw a further decrease in unit-wide rate of BPD to 19% (Figure 5). Balancing measures of rate of pneumothorax remained low at 3.2% throughout the project period for all infants admitted to the UMMMC NICU. Additionally, there was a decrease in the average number of invasive mechanical ventilation days per infant from 6.7 days per infant at baseline to 2.6 days per infant (Figure 6).

Respiratory outcomes are presented in Table 2. Significant decreases in BPD were seen after initiation of extending non-invasive ventilation until 32 0/7 weeks CGA and successful in incrementally reducing moderate and severe BPD from 38.4% to 15% (*p* < 0.0001). Following the initiation of a DR bundle guideline, the BPD rate decreased to 26.6% (*p* = 0.089). Subsequently, with implementation of the guideline for CPAP until 32 weeks CGA BPD rates further decreased to 23.3% representing a statistically significant difference from the baseline group (*p* = 0.011) and with revision of the surfactant guideline BPD rates were reduced to 15% (*p* < 0.0001) compared to baseline. After initiating our DR Bundle mechanical ventilation utilization was significantly reduced (*p* < 0.0001) and continued to further decline with subsequent PDSA cycles (*p* = 0.0025).

In logistic regression modeling of moderate and severe BPD to rule out potential association due to the differences in birth weights amongst cohorts, SGA status was not significantly associated. The differences in antenatal steroid utilization were also explored. Use of antenatal steroids was not significantly associated with moderate or severe BPD status and did not modify the significant differences in this outcome across intervention groups. Babies treated with inpatient steroids had 41.1 the odds (95% C.I.: 21.4–79.1) of developing moderate or severe BPD compared to babies who did not receive inpatient steroids.

## Discussion

In this work we describe our experiences with a multidisciplinary effort to decrease the incidence rate of BPD in our unit, whereas our unit's rates of BPD and among U.S. NICU's remains the most common morbidity of preterm infants. The interventions were derived from previously published literature on best practice for preventing and treating BPD and focused on use of non-invasive ventilation in the delivery room, early selective surfactant administration, early caffeine therapy, and continued non-invasive ventilation until 32 0/7 weeks CGA. Our unit was successful in implementing these guidelines, and the initiative achieved an impactful and sustained reduction in the incidence of BPD over the 5-year project period, representing an overall reduction of 56% without an increase in adverse outcomes.

Our results are reflective of real-world barriers and facilitators of implementing new guidelines. Our multidisciplinary BPD team worked together to research, inform, and create the guidelines. Additionally, our team educated and addressed the uncertainty staff members had around change to achieve adequate compliance with new guidelines.

A primary goal of our DR bundle was to optimize the delivery of non-invasive ventilation to avoid unnecessary need for mechanical ventilation. In clinical trials, mechanical ventilation has been linked in the development of BPD, so avoidance of invasive ventilation should correlate with lower rates of BPD in preterm infants (15, 16). Our protocol prioritizing noninvasive ventilation resulted in fewer infants in our cohort receiving invasive ventilation compared to baseline. Compliance rates reached or exceeded 90% for use of noninvasive ventilation in the DR. Despite rates of non-invasive ventilation in the delivery room of less than 100%, these rates may in fact have been the highest achievable rates, as a proportion of infants who received invasive mechanical ventilation in the delivery room and upon admission may have required mechanical ventilation per NRP guidelines and degree of illness and thus would not have qualified for non-invasive respiratory support. Our unit's utilization of mechanical ventilation has significantly decreased over the course of the project period, from a baseline of 6.7 days per infant to 2.6 days.

A recent systemic review by Healy et al. found that the most commonly utilized intervention in QI initiatives to decrease BPD included reducing mechanical ventilation by promoting early non-invasive ventilation (10). Like other single center QI initiatives, our group introduced a DR Bundle that promoted and initiated set guidelines that utilized non-invasive ventilation and specified respiratory modality and pressures based on birth gestational age (17, 18). Other centers have described use of early selective surfactant and early enteral caffeine delivery within the first 24 h of birth, but few others have combined all these initiatives in their described bundles (18). The DR Bundle was significant in reducing the use of mechanical ventilation but it was not sufficient in reducing rates of moderate and severe BPD, further interventions were the primary driver of reducing BPD in our cohort.

Beyond a DR Bundle that promoted early prevention of lung injury, our team also initiated guidelines that promoted maintenance on non-invasive ventilation until 32 0/7 weeks CGA (12). No other published QI guidelines have described standardized protocols to discontinue CPAP at a certain CGA in addition to weaning criteria. Our data suggests that this intervention led to further reduction in BPD incidence in our unit, specifically for those at highest risk for developing severe BPD.

Previous published QI initiatives were also mostly single center work that optimized non-invasive ventilation, early surfactant delivery, and caffeine utilization. Prior work failed to show significant improvement in infants at the highest risk, infants under 28 weeks’ gestation or with birth weights less than 1000 grams (10). Our guidelines applied to all infants below 32 weeks gestational age at birth, and were successful in reducing the severe BPD incidence rate by 60% from a baseline of 22%. In exploratory analysis of our cohort, SGA status at birth was not significantly associated with the development of BPD. Infants with severe BPD are at high risk for neurodevelopment impairment, and higher health care utilization rates post NICU discharge, including need for home oxygen and ventilator support, so reducing rates of severe BPD is particularly significant (10, 19, 20). Future QI initiatives may primarily focus on this cohort of infants to further reduce the rate of severe BPD (10, 20).

Despite high overall compliance rates, it is difficult to tease out which intervention contributed greatest or if the different interventions worked synergistically to improve our incidence rate of BPD. Our outcomes show the greatest reductions in BPD rates after the initiation of the guideline to extend non-invasive ventilation until 32 0/7 weeks CGA and was further reduced after revising our early selective surfactant guideline.

The limitations to our quality improvement work are that it reflects practice change guidelines in a single Level III NICU, some changes of which may not be broadly generalizable to other units. Table 1 highlights one significant difference in birth weights between two cohort groups, but not overall. The rate of infants that are small for gestational age in our unit may differ from other units and may be an independent risk factor for BPD development. However, in logistic regression, being small for gestational age was not significantly associated with moderate or severe BPD status and did not modify interventional group associations with this outcome. Further, there were other non-respiratory management changes such as standardizing use of antenatal steroids for mothers at risk of preterm labor. Improvements in more mothers receiving full course of antenatal steroids came after an update in the opinion by the American College of Obstetricians and Gynecologists in late 2017 (21) and our OBGYN Departments response and QI work. This improvement is demonstrated by the increased utilization of full-course antenatal steroids after Q4 2017 and an observed decrease in our unit's utilization of post-natal steroids. However, the differences in antenatal steroid use were not significantly associated with moderate or severe BPD in infants. Further research would need to evaluate the significance and correlation of the observed trends.

## Conclusion

Advances in neonatal care have led to higher amounts of infants developing BPD and reducing the incidence of BPD has become a primary focus of neonatal units. In this paper, we highlight our multidisciplinary team's QI work in reducing our units BPD incidence. Implementing a DR Bundle, continuing non-invasive ventilation until 32 0/7 weeks gestation, and revising early surfactant delivery guidelines helped reduce our overall BPD rate by 56% and our severe BPD rate by 60% over the 5-year project duration.

## Data Availability

The raw data supporting the conclusions of this article will be made available by the authors, without undue reservation.
