# Supplementary figures and images for: Respiratory support strategies in the prevention of bronchopulmonary dysplasia: A single center quality improvement initiative
Source: Front Pediatr. 2022 Dec 12;10:1012655. doi: 10.3389/fped.2022.1012655 (PMC9790967; doi:10.3389/fped.2022.1012655)

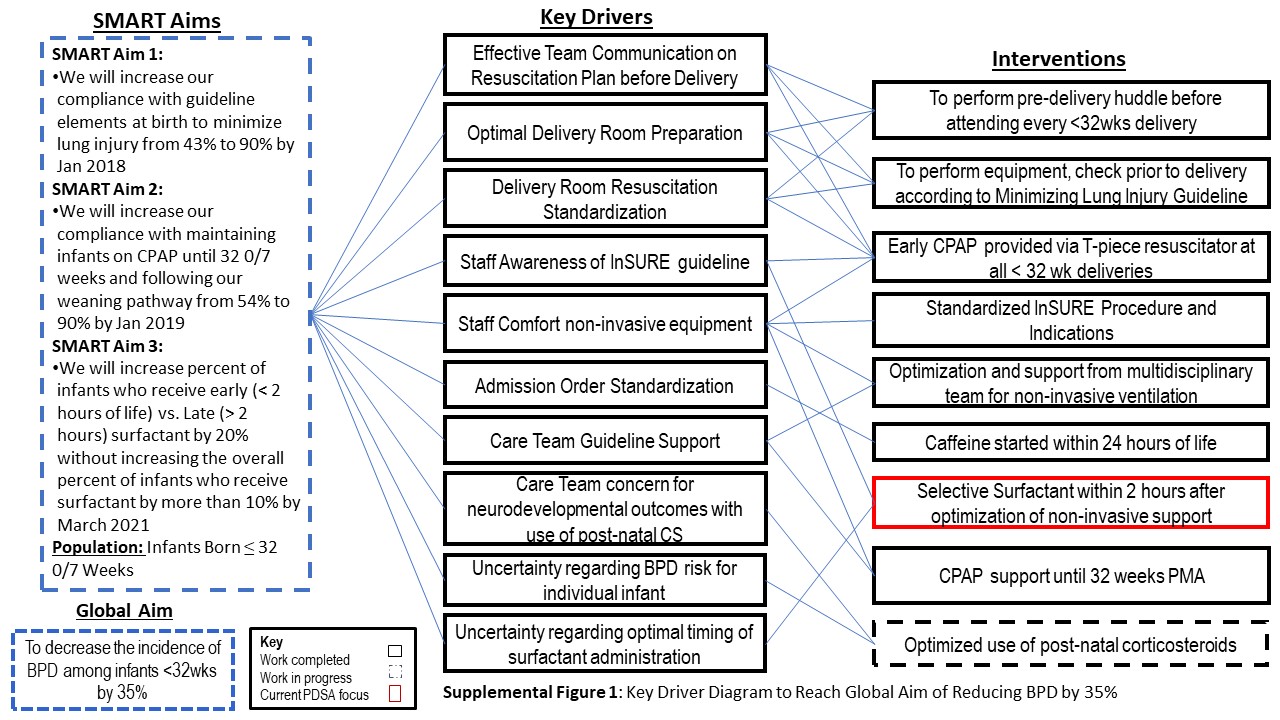

Supplement: Supplementary file 1 [file Image1.jpeg]

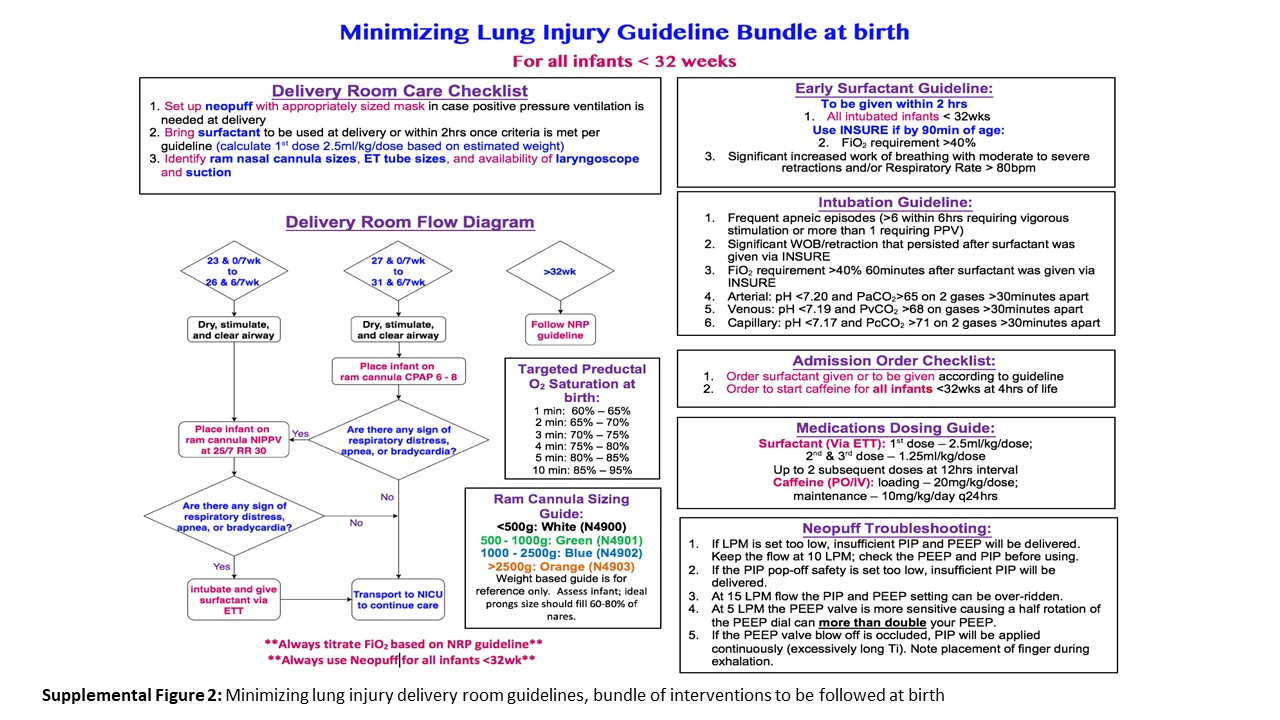

Supplement: Supplementary file 2 [file Image2.jpeg]

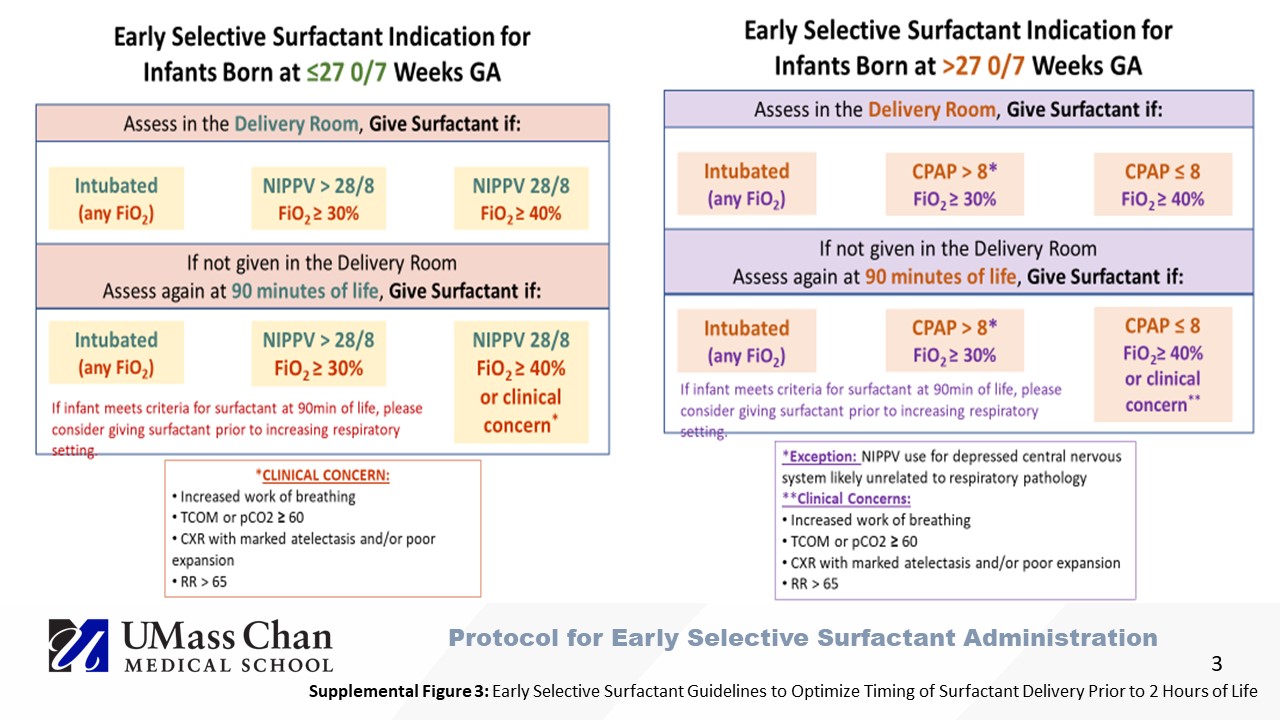

Supplement: Supplementary file 3 [file Image3.jpeg]
